# Characterization of the human helicotrema: implications for cochlear duct length and frequency mapping

**DOI:** 10.1186/s40463-019-0398-8

**Published:** 2020-01-06

**Authors:** Luke Helpard, Hao Li, Helge Rask-Andersen, Hanif M. Ladak, Sumit K. Agrawal

**Affiliations:** 1 School of Biomedical Engineering, Western University, London, Ontario Canada; 2 Department of Surgical Sciences, Head and Neck Surgery, Section of Otolaryngology, Uppsala University Hospital, Uppsala, Sweden; 3 Department of Otolaryngology, Uppsala University Hospital, Uppsala, Sweden; 4 Department of Otolaryngology - Head and Neck Surgery, Western University, London, Ontario Canada; 5 Department of Medical Biophysics, Western University, London, Ontario Canada; 6 Department of Electrical and Computer Engineering, Western University, London, Ontario Canada

**Keywords:** Helicotrema, Helicotrema size, Cochlear duct length, Basilar membrane, Cochlear apex, Cochlear implant, Frequency mapping, Synchrotron radiation

## Abstract

**Background:**

Despite significant anatomical variation amongst patients, cochlear implant frequency-mapping has traditionally followed a patient-independent approach. Basilar membrane (BM) length is required for patient-specific frequency-mapping, however cochlear duct length (CDL) measurements generally extend to the apical tip of the entire cochlea or have no clearly defined end-point. By characterizing the length between the end of the BM and the apical tip of the entire cochlea (helicotrema length), current CDL models can be corrected to obtain the appropriate BM length. Synchrotron radiation phase-contrast imaging has made this analysis possible due to the soft-tissue contrast through the entire cochlear apex.

**Methods:**

Helicotrema linear length and helicotrema angular length measurements were performed on synchrotron radiation phase-contrast imaging data of 14 cadaveric human cochleae. On a sub-set of six samples, the CDL to the apical tip of the entire cochlea (CDL_TIP_) and the BM length (CDL_BM_) were determined. Regression analysis was performed to assess the relationship between CDL_TIP_ and CDL_BM_.

**Results:**

The mean helicotrema linear length and helicotrema angular length values were 1.6 ± 0.9 mm and 67.8 ± 37.9 degrees, respectively. Regression analysis revealed the following relationship between CDL_TIP_ and CDL_BM_: CDL_BM_ = 0.88(CDL_TIP_) + 3.71 (*R*^2^ = 0.995).

**Conclusion:**

This is the first known study to characterize the length of the helicotrema in the context of CDL measurements. It was determined that the distance between the end of the BM and the tip of the entire cochlea is clinically consequential. A relationship was determined that can predict the BM length of an individual patient based on their respective CDL measured to the apical tip of the cochlea.

## Background

Cochlear implants (CI) consist of an electrode array that is inserted along the cochlea, with discrete contacts providing stimulus directly to the auditory nerve to produce the sensation of sound. Despite significant anatomical variation in the cochleae of patients [1–4], CI frequency-mapping has traditionally followed a patient-independent approach, which may affect patient outcomes. If the cochlear duct length (CDL) can be determined pre-operatively, an appropriate length CI can be selected, and the pitch-map of this implant can be customized using Greenwood’s equation [5] if the final electrode locations are determined through post-operative imaging. If patient-specific pitch-maps are determined, CI electrode arrays can be programmed to match the true tonotopic arrangement of an individual patients’ cochlea [6, 7]. Preliminary evidence suggests that this may result in improved hearing outcomes through music appreciation, pitch-discernment, and speech perception [7, 8].

Investigators have used several manual and analytical approaches to estimate CDL on an individual level to move towards customized CI programming [3, 9–13]. Manual methods rely on the placement of points along the entire length of the cochlea, and although this technique yields accurate results for research purposes, it is not feasible in a clinical setting [4, 11, 14, 15]. Analytical approaches, such as the *A* value technique, utilize models developed from reference cochleae and generally use a small collection of measurements to estimate CDL [10, 12, 13, 16]. To achieve clinically relevant results using either technique, anatomically accurate boundary conditions must be in place to determine the start- and end-point of the CDL measurements [17]. Although the round window (RW) has been ubiquitously used as the CDL measurement start-point, lack of visual clarity in the cochlear apex has caused ambiguity in the end-point for measurements at the cochlear apex. In addition, the cochlear apex is highly variable, and therefore many modelling techniques, such as the *A* value technique, have only been accurate up to the cochlear two-turn length [13, 16, 18]. Clinically measuring the cochlear two-turn length using the *A* value can be useful for surgical planning and specifying electrode lengths, however, the entire basilar membrane (BM) length including the apical turn is needed to utilize Greenwood’s equation.

The helicotrema is the most apical portion of the cochlea and is defined as the region where the scala tympani and scala vestibuli meet at the end of the BM. The BM narrows in its most apical portion, and a visible gap can be observed in the helicotrema from the end of the BM to the apical tip of the entire cochlea [19, 20]. BM length is required for the use of Greenwood’s equation, however CDL measurements often extend to the apical tip of the entire cochlea or have no clearly defined end-point [4, 14, 15]. This is because the BM is often not visible at the helicotrema, even when using high-resolution micro-CT techniques [2]. Correction factors have been proposed to relate CDL measurements at the lateral wall (LW) to those at the organ of Corti, however these only correct for the radial location of the measurement and not for the helicotrema length [13, 18]. In addition to CDL, more accurate representations of the helicotrema are required for the development of numerical models to study the biophysics of the cochlear apex [21]. Synchrotron radiation phase-contrast imaging (SR-PCI) is a novel imaging approach that yields higher soft-tissue contrast than comparable techniques such as micro-CT [18, 22, 23]. In SR-PCI data, the BM is visible and can be measured in detail through its entire length to the helicotrema. In contrast to histologic sectioning, SR-PCI allows for three-dimensional (3D) volume reconstructions and does not require sectioning, decalcification, staining, and slide mounting [22].

To our knowledge, no geometric analysis has been conducted on the helicotrema region in the context of CDL measurements. The objective of this study was to characterize the length between the end of the BM and the tip of the cochlea using SR-PCI data of cadaveric human cochleae.

## Methods

### Sample preparation and scanning

All cadaveric specimens used in this study were obtained with permission from the body bequeathal program at Western University (London, ON, Canada) in accordance with the Anatomy Act of Ontario and Western’s Committee for Cadaveric Use in Research (approval #19062014). The entire Western University synchrotron database was analysed, and specimens were included if they contained an intact BM throughout the entire apical turn. SR-PCI data from 14 cadaveric human cochleae were included in this study.

All samples were scanned at the Canadian Light Source Inc. (Saskatoon, SK, Canada) using the Biomedical Imaging and Therapy beamline (05ID-2). The detector had an effective pixel size of 9 μm (isotropic), and 3000 projections were acquired over 180-degrees of sample rotation. Specifications of sample preparation and of the imaging technique have been previously reported [18, 22, 23].

### Helicotrema measurements

The helicotrema linear length (HLL) and helicotrema angular length (HAL) were measured on all 14 samples. HLL is defined as the linear distance (measured in mm) from the end of the BM to the apical tip of the cochlea along the LW. With the modiolus used as the axis of rotation, the HAL is defined as the angular length (measured in degrees) from the end of the BM to the apical tip of the cochlea along the LW.

**Fig. 1:**
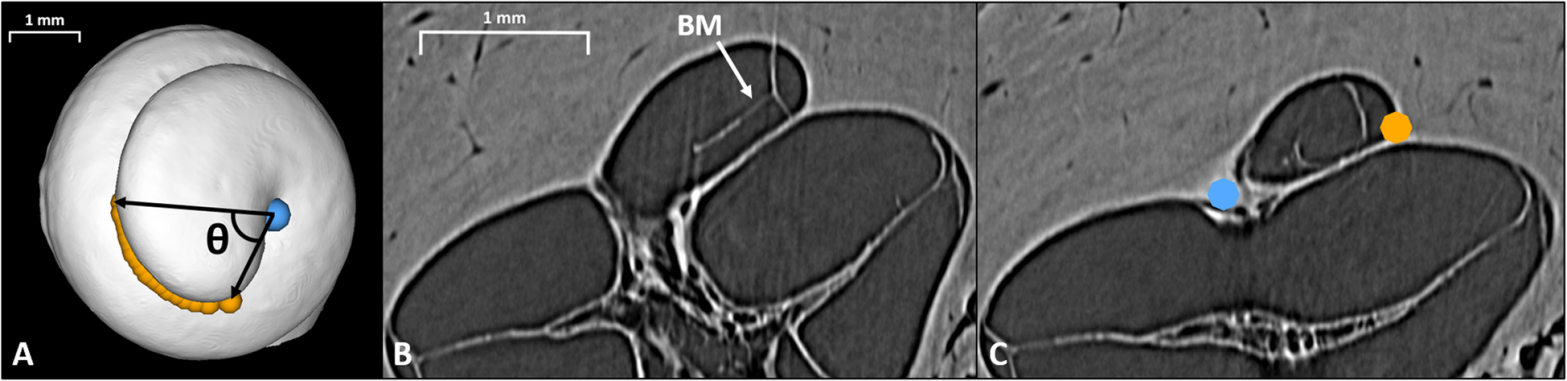
**a** Illustration of the fiducials placed along the LW from the end of the BM to the apical tip of the cochlea (orange) on a 3D model of the middle and apical turns. The fiducial placed at the modiolar axis is additionally visible (blue). ϴ is a visual representation of the HAL. **b** A cross-sectional image slice from SR-PCI data. The BM is visible and annotated. When progressing apically from this slice, the BM collapses towards the middle turn and quickly reaches its end-point. **c** A cross-sectional slice after the BM end-point. Fiducials are seen along the LW at the approximate level of the BM end-point (orange), and at the modiolar axis location (blue). LW denotes lateral wall; HAL denotes helicotrema angular length; SR-PCI denotes synchrotron radiation phase-contrast imaging; BM denotes basilar membrane

**Fig. 2:**
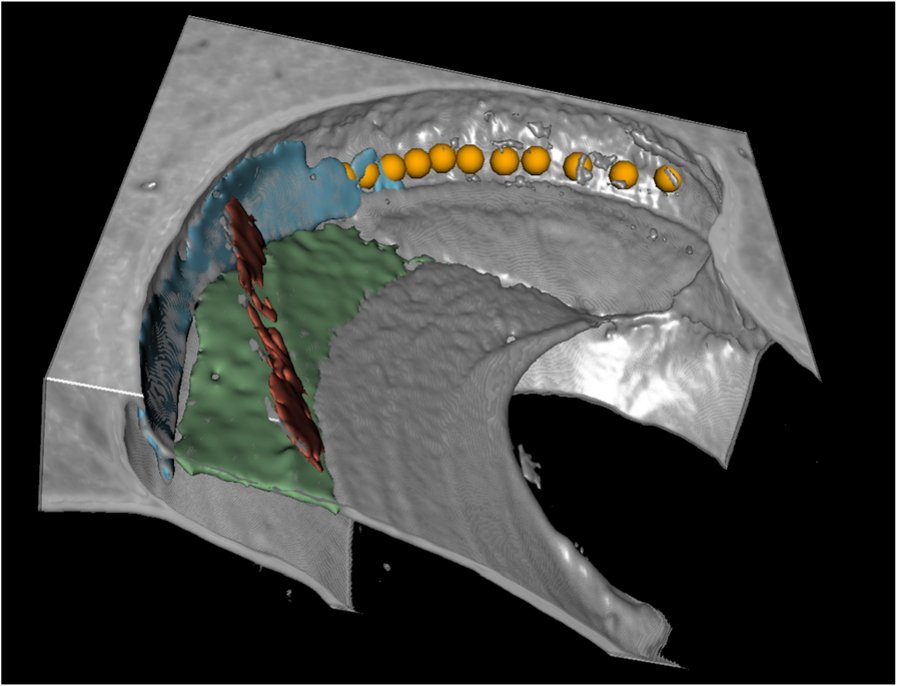
3D volume rendering of the cochlear apex. Fiducials (orange) are placed along the LW from the end of the BM to the tip of the cochlea. The BM is coloured in green, Reissner’s membrane is coloured in red, and the spiral ligament is coloured in blue. LW denotes lateral wall; BM denotes basilar membrane

To measure the HLL and HAL, fiducials were placed along the LW of the cochleae at the level of the BM in the helicotrema region using 3D Slicer (https://www.slicer.org/), as illustrated in Fig. 1. The start-point for the helicotrema measurements was at the end of the BM, indicated by the point where the BM was no longer visible between the osseous spiral lamina and the spiral ligament in image slices and 3D volume renderings. An example start-point for helicotrema measurements is displayed using a volume rendering in Fig. 2. The end-point for the helicotrema measurements was at the apical tip of the cochlea, indicated as the point where the LW reached its vertex and began to turn towards the modiolus (as illustrated in Fig. 1). Image slices in a plane orthogonal to the cochlear scalae path were used to place fiducials from the end of the BM to the apical tip of the cochlea. Lastly, a fiducial was placed at the estimated modiolar axis location in the same plane as the previously described set of LW fiducials.

### Image processing

Prior to the measurement of the HLL and HAL values, a custom Python (https://www.python.org/) script was used to process the helicotrema fiducials. To eliminate variance in the measurements due to inconsistencies in the level of fiducial placement on the LW, a plane of best fit was determined amongst the helicotrema fiducials. All fiducials were then orthogonally projected into the plane of best fit prior to measurement. The HLL was determined by summing the Euclidean distances between subsequent fiducials. The HAL was determined by first defining vectors between the modiolar axis fiducial and each helicotrema fiducial, and then summing the angles between subsequent fiducials to provide accuracy in three-dimensions.

### CDL measurements

CDL values were obtained for a sub-set of six samples (out of the total 14 samples) that had visible RWs in the SR-PCI dataset. Using image slices and 3D volume renderings, fiducials were placed along the entire length of the cochleae. With the modiolar axis used as the axis of rotation and the RW serving as the 0-degree point, fiducials were placed on the LW at approximately 30-degree intervals from the RW to the apical tip of the entire cochlea. The last fiducial was placed directly at the apical tip of the cochleae. CDL values were then determined by summing the Euclidean distances between subsequent fiducials. CDL measured to the most apical tip of the entire cochlea is defined as CDL_TIP_.

BM length was estimated in these six samples by subtracting the HLL from the respective CDL_TIP_ value. BM length is defined as CDL_BM_.

### Statistical analyses

The mean and standard deviation were calculated for the HLL and HAL values. *Mean ± standard deviation* is the convention used throughout when presenting data. Kolmogorov-Smirnov tests were conducted to assess normality in the HLL and HAL values. Confidence intervals were constructed for the HLL and HAL values at the 95% level using a *t*-distribution. Linear regression was performed to determine the relationship between HLL and HAL. This regression result was used to determine if there was a consistent morphology (helical shape) observed across samples in the helicotrema region.

The mean and standard deviation were calculated for the CDL_TIP_ and CDL_BM_ values in the sub-set of samples that had visible RW membranes. A linear regression was performed to determine the relationship between CDL_TIP_ and CDL_BM_. This regression result was used to estimate the correction factor required to retrieve CDL_BM_ from CDL_TIP_ measurements.

All statistical analyses were completed using MATLAB (version R2018, The MathWorks, Inc., Natick, MA).

## Results

### HLL and HAL measurements

HLL and HAL values were measured on all 14 SR-PCI samples. The mean HLL was 1.6 ± 0.9 mm (95% confidence interval: [1.1 mm, 2.1 mm]), with minimum and maximum values of 0.7 mm and 3.8 mm, respectively. The mean HAL was 67.8 ± 37.9 degrees (95% confidence interval: [46.0 degrees, 89.7 degrees]), with minimum and maximum values of 32.4 degrees and 175.9 degrees, respectively.

**Fig. 3:**
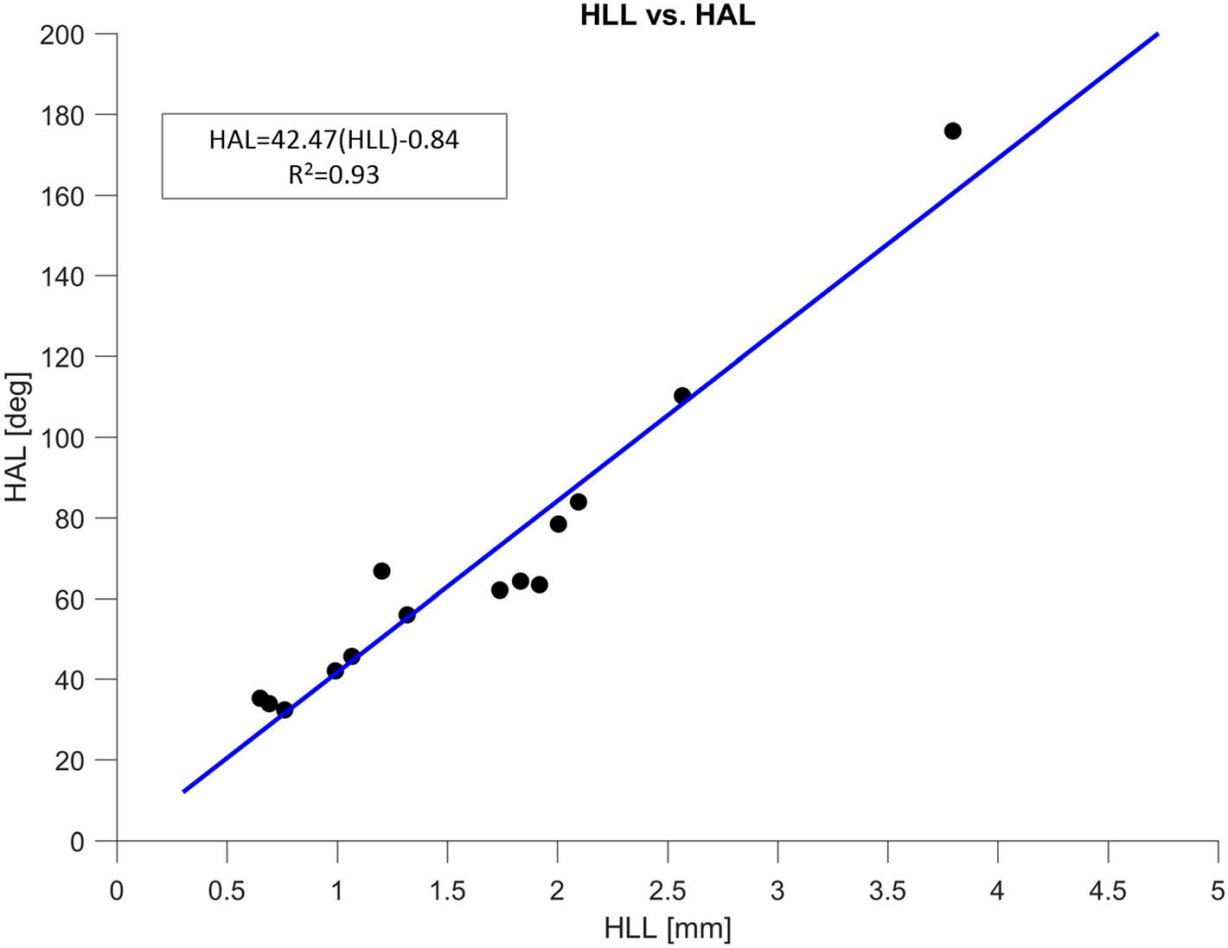
Plot displaying the regression result between HLL and HAL. Individual pairs of HLL and HAL values are plotted as points, and the linear curve of best fit is illustrated in blue. HLL denotes helicotrema linear length; HAL denotes helicotrema angular length

Regression analysis was performed to determine the relationship between the 14 HLL and HAL measurements. Regression analysis yielded the equation, HLL = 42.47(HAL)–0.84 (*R*^2^ = 0.933). The determined linear function and the individual pairs of HLL and HAL values are illustrated in the plot in Fig. 3.

### CDL measurements

For the six samples that had intact BMs in the apex and visible RW membranes in the SR-PCI data, the CDL_TIP_ was directly measured. The mean CDL_TIP_ value measured was 39.9 ± 1.7 mm. In these six samples, the CDL_BM_ was determined by subtracting the HLL value from the CDL_TIP_ value. The mean CDL_BM_ was determined to be 39.0 ± 1.5 mm.

**Fig. 4:**
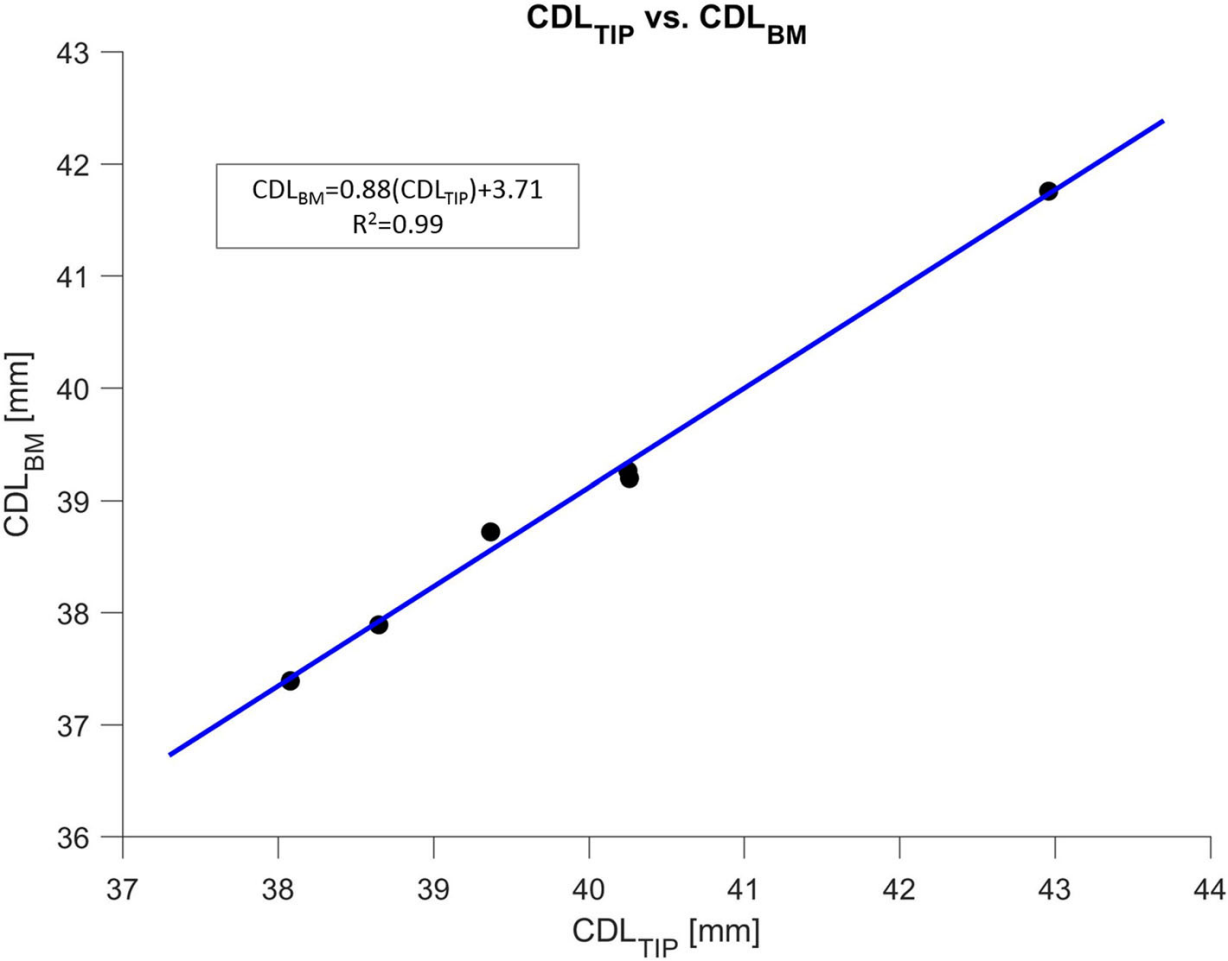
Plot displaying the regression result between CDL_TIP_ and CDL_BM_. Individual pairs of CDL_TIP_ and CDL_BM_ values are plotted as points, and the linear curve of best fit is illustrated in blue. CDL_TIP_ denotes length measured to the apical tip of the cochlea; CDL_BM_ denotes BM length

Regression analysis was conducted to determine the relationship between CDL_TIP_ and CDL_BM_ in the six samples. The regression analysis yielded the equation, CDL_BM_ = 0.88(CDL_TIP_) + 3.71 (*R*^2^ = 0.995). The determined linear function and the individual pairs of CDL_TIP_ and CDL_BM_ values are illustrated in the plot in Fig. 4.

## Discussion

CDL measurement has been a topic of growing attention in the literature, largely due to the interest in customizing CI pitch-maps for individual patients using Greenwood’s equation [5, 7, 8]. Greenwood’s equation uses an exponential function to model the frequency distribution of individual cochleae based on the respective CDL_BM_. There has, however, been a gap between the requirement of CDL_BM_ for Greenwood’s equation and the length measurements possible using currently available imaging techniques. Due to low discernment of the BM in the apical region of the cochlea, current CDL models generally extend to the most apical point of the entire cochlea or have no clearly defined end-point in the helicotrema region.

Both direct measurements and analytical estimates are subject to error due to ambiguities in the helicotrema region. Avci et al. produced a detailed analysis of the cochlear scalae using high-resolution micro-CT [2]. Unfortunately, the authors were unable to characterize the soft-tissue beyond the middle turn, and therefore the cochlear apex was excluded from the analysis. Direct length measurement techniques, such as those proposed by Vu et al. [24], require the user to identify the exact end-point of the BM to use Greenwood’s equation. This is generally not achievable due to the lack of visualization of soft-tissue membranes in the apex. The most novel analytical estimates, such as those proposed by Schurzig et al. [25], require the user to specify an angular position for which they would like to estimate the length. In this case, the exact angular length of the BM must be identified for Greenwood’s equation to be used.

The mean HLL value in our dataset was 1.6 mm, and a maximum value of 3.8 mm was observed. Additionally, CDL_BM_ was observed to be significantly shorter than the CDL_TIP._ These results indicate that the assumption that the BM reaches the end of the entire cochlea could be clinically consequential. When publishing the function for the cochlear tonotopic map, Greenwood presented the function coefficients specifically for a cochlea with a CDL_BM_ of 35 mm [5]. In order to determine the frequency map for a cochlea with a different CDL_BM_, the coefficients must be scaled appropriately, as described by Greenwood [5]. Utilizing the mean values determined in this work, we examine the hypothetical case of a cochlea with CDL_BM_ of 39.0 mm and HLL of 1.6 mm. It was found that the assumption that the BM reaches the very apical tip of the cochlea (error of 1.6 mm) could result in a pitch-mismatch of approximately 280 Hz at an insertion depth of 5 mm, and approximately 120 Hz at an insertion depth of 25 mm. A 120 Hz mismatch at a 25 mm insertion depth (approximately 780 Hz–900 Hz) corresponds to a gap of over two semitones, and this pitch-mismatch can potentially be important for the perception of music, complex speech, and tonal languages [26, 27]. CIs currently have limitations due to their insertion depth and the resolution of electrode stimulation, however this hypothetical situation provides evidence that the size of the helicotrema has a measurable effect on the frequency distribution and perception in individuals. Generalized CI pitch-maps have been reported to result in a pitch-mismatch of over one octave [28, 29], due to variance in cochlear size and difficulties measuring the BM in the helicotrema and hook region. Accurate modelling of the helicotrema has the potential to reduce a portion of this error. The cochlear hook region is another complex portion of the BM that has had limited description previously in the literature. Current measurement protocols in the hook region can also result in CDL errors in the order of 2 mm, and to further reduce CDL errors and consequently pitch-mapping errors, a complete analysis of the cochlear hook region in three-dimensions is required. Tonotopic frequency mismatch is potentially consequential because it is suggested that correct tonotopic stimulation is required for complex-sound perception [30].

The high correlation observed between HLL and HAL measurements indicate a consistent morphological relationship in the helicotrema region. A linear relationship was determined that significantly predicted the HAL of the cochleae based on the HLL, with changes in HLL accounting for over 93% of the variability detected in the HAL. This may imply that cochleae twist in a consistent way in the helicotrema region regardless of their size. This is a valuable preliminary result because it indicates that the apical turns of the cochlea have a predictable behaviour.

Regression analysis additionally revealed a significant relationship between CDL_TIP_ and CDL_BM_ in our sample set. It was observed that CDL_BM_ can be estimated with confidence based on the CDL_TIP_ value using the equation: CDL_BM_ = 0.88(CDL_TIP_) + 3.71. CDL_BM_ is not directly measurable on most clinical and research imaging modalities, however CDL_TIP_ is a parameter that can be estimated using validated published techniques and models [10]. This study, therefore, provides a correction factor to accurately predict the total CDL_BM_ based on CDL_TIP_. This can be incorporated into various analytical equations already published [10, 13, 18, 25]. Furthermore, the HLL and HAL values presented herein can be used to develop more anatomically accurate numerical models of the human cochlea. Current models have made approximations regarding the size of the helicotrema, and the detailed measurements from this study can be integrated to produce more accurate biomechanical models of the cochlear apex [21, 31].

The sample size in this study was limited by the challenges associated with SR-PCI. SR-PCI was conducted at the Canadian Light Source Inc., a government facility that competitively grants scan time. The synchrotron facility is geographically distant from the home institution of the authors, and access is limited based on the high facility demand. The initial cochlear dataset scanned with SR-PCI was chosen to represent a wide distribution of CDL values and cochlear shapes to maximize representation of the general population. The relationships derived in this study are limited by the small sample size, however they provide statistically strong trends that are expected to relate CDL_TIP_ to CDL_BM_ in patients. Furthermore, measurements in this study were taken at the LW of the cochleae. Previous publications have suggested the organ of Corti length is clinically relevant, and suggest correction factors to relate CDL values at the LW to CDL values at the organ of Corti [32]. These previously published correction factors can be used concurrently with the adjustment presented herein to achieve CDL_BM_ at different locations on the cochlear partition. This was the first study to analyze the length and morphology of the helicotrema and cochlear apex. Future studies will include additional SR-PCI scans of the helicotrema region with a more dedicated field of view to optimize visualization of anatomic detail. Electron microscope scans of the cochlear apex will be combined with the SR-PCI findings to obtain accurate 3D models of the BM and its supporting structures in the helicotrema region.

## Conclusion

Although morphological analyses were previously attempted [2], SR-PCI allowed high-resolution imaging of both the cochlear soft tissues and bony walls in the apex. HLL and HAL values were measured on 14 cadaveric SR-PCI scans, and additionally CDL_TIP_ and CDL_BM_ were determined on a sub-set of six samples. It was determined that a significant relationship exists between HLL and HAL in our sample set, indicating constant morphology in the helicotrema region. A significant relationship was also found between CDL_BM_ and CDL_TIP_ in our sample set. This indicates that CDL_BM_ can be estimated from CDL_TIP_ measurements using the equations presented herein. CDL_BM_ can be subsequently used for more anatomically accurate CI planning and pitch-mapping.

## Data Availability

The datasets analyzed during the current study are available upon reasonable request at the discretion of the authors.

## Abbreviations

3D: Three-dimensional
BM: Basilar membrane
CDL: Cochlear duct length
CI: Cochlear implant
HAL: Helicotrema angular length
HLL: Helicotrema linear length
LW: Lateral wall
RW: Round window
SR-PCI: Synchrotron radiation phase-contrast imaging
